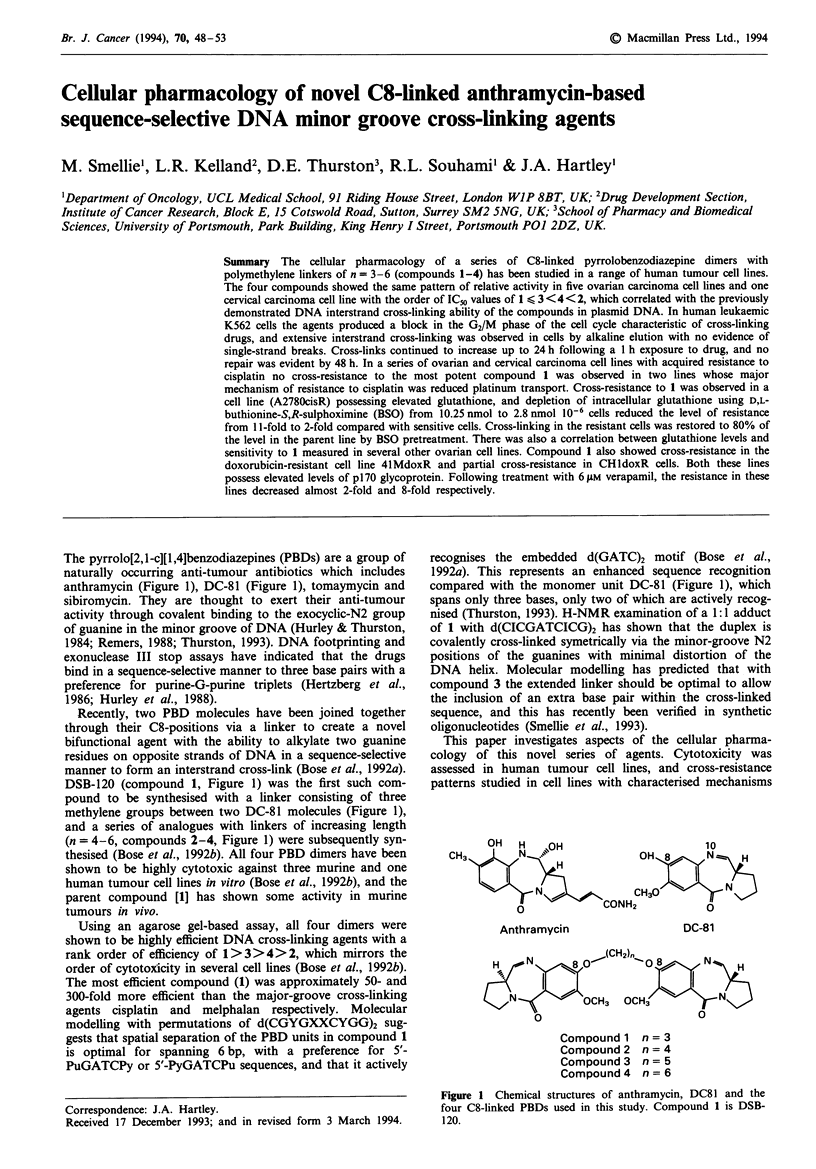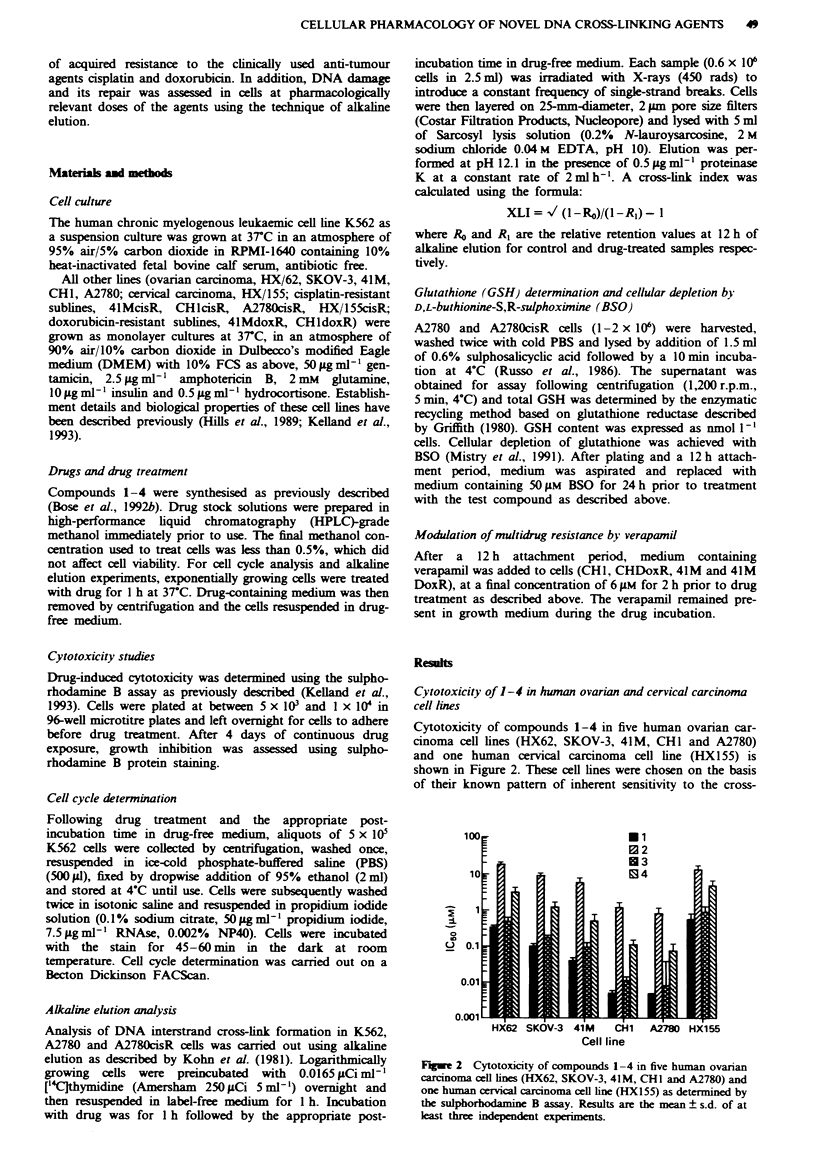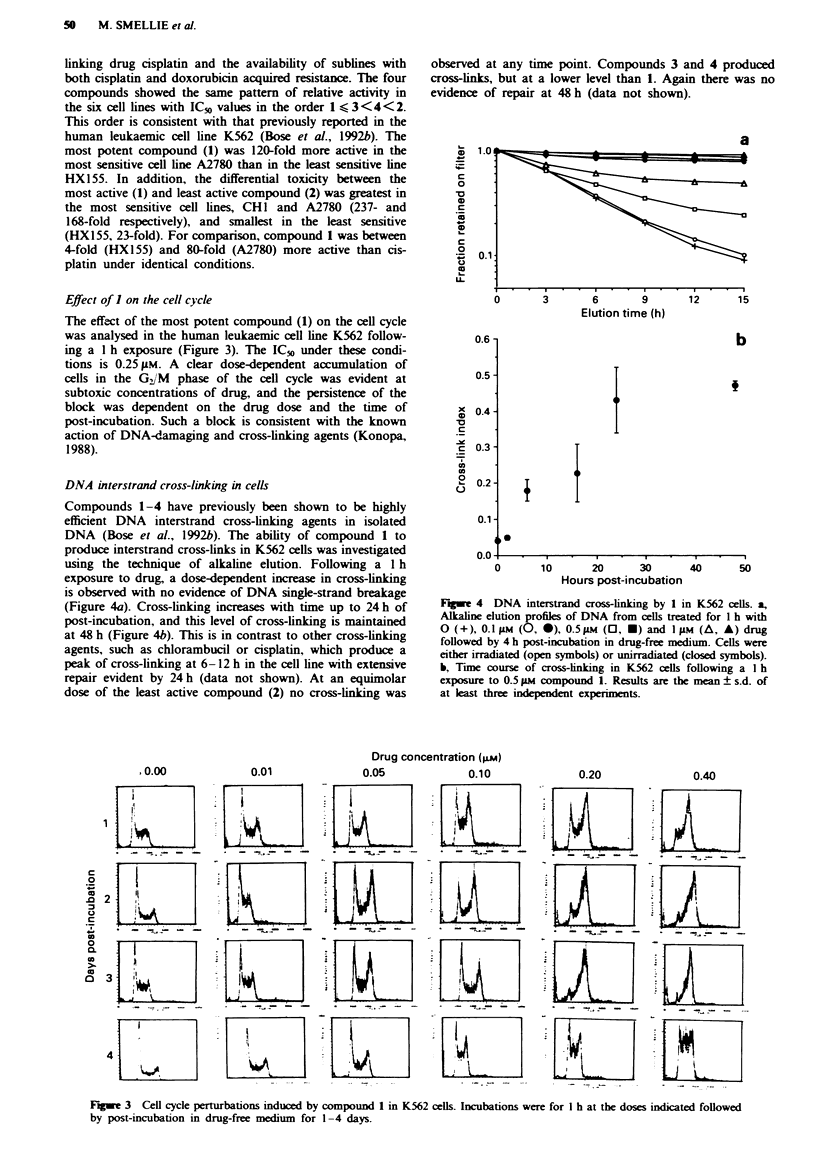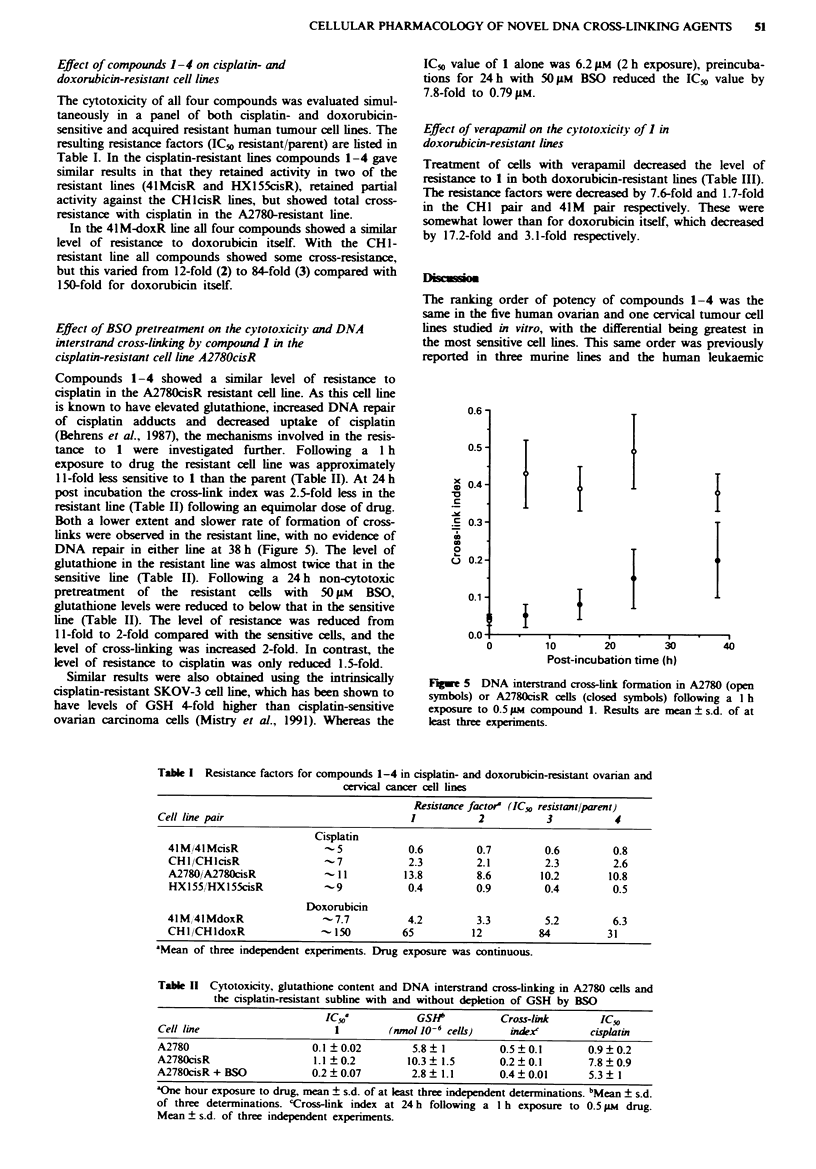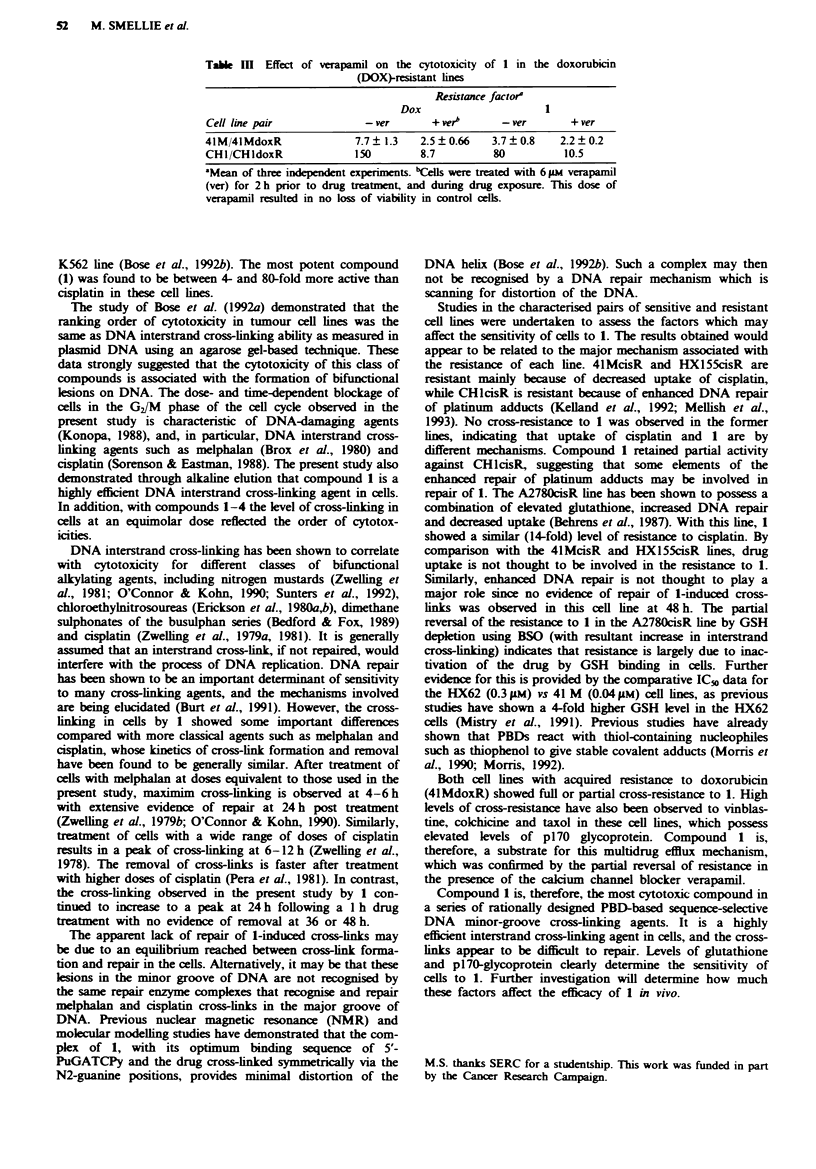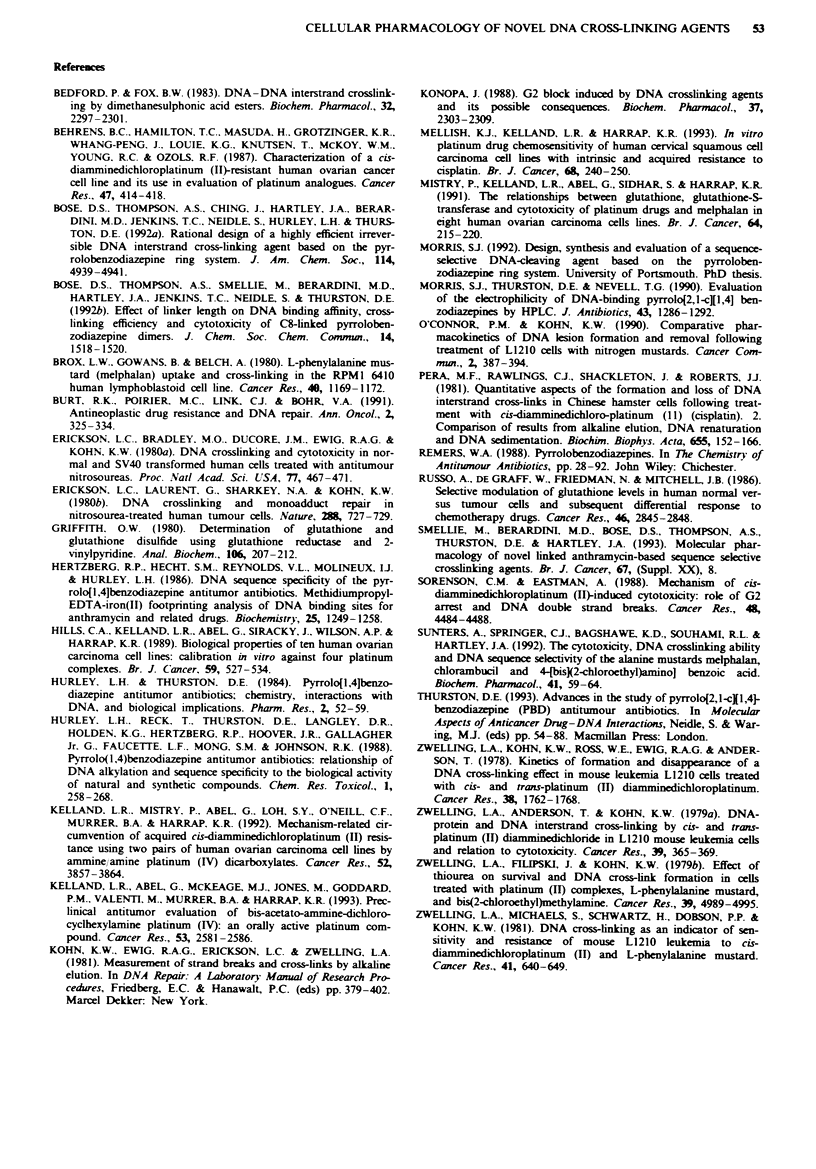# Cellular pharmacology of novel C8-linked anthramycin-based sequence-selective DNA minor groove cross-linking agents.

**DOI:** 10.1038/bjc.1994.248

**Published:** 1994-07

**Authors:** M. Smellie, L. R. Kelland, D. E. Thurston, R. L. Souhami, J. A. Hartley

**Affiliations:** Department of Oncology, UCL Medical School, London, UK.

## Abstract

The cellular pharmacology of a series of C8-linked pyrrolobenzodiazepine dimers with polymethylene linkers of n = 3-6 (compounds 1-4) has been studied in a range of human tumour cell lines. The four compounds showed the same pattern of relative activity in five ovarian carcinoma cell lines and one cervical carcinoma cell line with the order of IC50 values of 1 < or = 3 < 4 < 2, which correlated with the previously demonstrated DNA interstrand cross-linking ability of the compounds in plasmid DNA. In human leukaemic K562 cells the agents produced a block in the G2/M phase of the cell cycle characteristic of cross-linking drugs, and extensive interstrand cross-linking was observed in cells by alkaline elution with no evidence of single-strand breaks. Cross-links continued to increase up to 24 h following a 1 h exposure to drug, and no repair was evident by 48 h. In a series of ovarian and cervical carcinoma cell lines with acquired resistance to cisplatin no cross-resistance to the most potent compound 1 was observed in two lines whose major mechanism of resistance to cisplatin was reduced platinum transport. Cross-resistance to 1 was observed in a cell line (A2780cisR) possessing elevated glutathione, and depletion of intracellular glutathione using D,L-buthionine-S,R-sulphoximine (BSO) from 10.25 nmol to 2.8 nmol 10(-6) cells reduced the level of resistance from 11-fold to 2-fold compared with sensitive cells. Cross-linking in the resistant cells was restored to 80% of the level in the parent line by BSO pretreatment. There was also a correlation between glutathione levels and sensitivity to 1 measured in several other ovarian cell lines. Compound 1 also showed cross-resistance in the doxorubicin-resistant cell line 41MdoxR and partial cross-resistance in CH1doxR cells. Both these lines possess elevated levels of p170 glycoprotein. Following treatment with 6 microM verapamil, the resistance in these lines decreased almost 2-fold and 8-fold respectively.


### Cellular pharmacology of novel C8-linked anthramycin-based sequence-selective DNA minor groove cross-linking agents

M. Smellie[1], L.R. Kelland[2], D.E. Thurston[3], R.L. Souhami[1] & J.A. Hartley[1]

[1]*Department of Oncology, UCL Medical School, 91 Riding House Street, London W1P 8BT, UK;* [2]*Drug Development Section, Institute of Cancer Research, Block E, 15 Cotswold Road, Sutton, Surrey SM2 5NG, UK;* [3]*School of Pharmacy and Biomedical Sciences, University of Portsmouth, Park Building, King Henry I Street, Portsmouth PO1 2DZ, UK.*

**Summary** The cellular pharmacology of a series of C8-linked pyrrolobenzodiazepine dimers with polymethylene linkers of $n = 3-6$ (compounds **1-4**) has been studied in a range of human tumour cell lines. The four compounds showed the same pattern of relative activity in five ovarian carcinoma cell lines and one cervical carcinoma cell line with the order of $IC_{50}$ values of **1** $\leqslant$ **3** < **4** < **2**, which correlated with the previously demonstrated DNA interstrand cross-linking ability of the compounds in plasmid DNA. In human leukaemic K562 cells the agents produced a block in the $G_2/M$ phase of the cell cycle characteristic of cross-linking drugs, and extensive interstrand cross-linking was observed in cells by alkaline elution with no evidence of single-strand breaks. Cross-links continued to increase up to 24 h following a 1 h exposure to drug, and no repair was evident by 48 h. In a series of ovarian and cervical carcinoma cell lines with acquired resistance to cisplatin no cross-resistance to the most potent compound **1** was observed in two lines whose major mechanism of resistance to cisplatin was reduced platinum transport. Cross-resistance to **1** was observed in a cell line (A2780cisR) possessing elevated glutathione, and depletion of intracellular glutathione using D,L-buthionine-*S*,*R*-sulphoximine (BSO) from 10.25 nmol to 2.8 nmol $10^{-6}$ cells reduced the level of resistance from 11-fold to 2-fold compared with sensitive cells. Cross-linking in the resistant cells was restored to 80% of the level in the parent line by BSO pretreatment. There was also a correlation between glutathione levels and sensitivity to **1** measured in several other ovarian cell lines. Compound **1** also showed cross-resistance in the doxorubicin-resistant cell line 41MdoxR and partial cross-resistance in CH1doxR cells. Both these lines possess elevated levels of p170 glycoprotein. Following treatment with 6 μM verapamil, the resistance in these lines decreased almost 2-fold and 8-fold respectively.

The pyrrolo[2,1-c][1,4]benzodiazepines (PBDs) are a group of naturally occurring anti-tumour antibiotics which includes anthramycin (Figure 1), DC-81 (Figure 1), tomaymycin and sibiromycin. They are thought to exert their anti-tumour activity through covalent binding to the exocyclic-N2 group of guanine in the minor groove of DNA (Hurley & Thurston, 1984; Remers, 1988; Thurston, 1993). DNA footprinting and exonuclease III stop assays have indicated that the drugs bind in a sequence-selective manner to three base pairs with a preference for purine-G-purine triplets (Hertzberg *et al.*, 1986; Hurley *et al.*, 1988).

Recently, two PBD molecules have been joined together through their C8-positions via a linker to create a novel bifunctional agent with the ability to alkylate two guanine residues on opposite strands of DNA in a sequence-selective manner to form an interstrand cross-link (Bose *et al.*, 1992*a*). DSB-120 (compound **1**, Figure 1) was the first such compound to be synthesised with a linker consisting of three methylene groups between two DC-81 molecules (Figure 1), and a series of analogues with linkers of increasing length ($n = 4-6$, compounds **2-4**, Figure 1) were subsequently synthesised (Bose *et al.*, 1992*b*). All four PBD dimers have been shown to be highly cytotoxic against three murine and one human tumour cell lines *in vitro* (Bose *et al.*, 1992*b*), and the parent compound [**1**] has shown some activity in murine tumours *in vivo*.

Using an agarose gel-based assay, all four dimers were shown to be highly efficient DNA cross-linking agents with a rank order of efficiency of **1** > **3** > **4** > **2**, which mirrors the order of cytotoxicity in several cell lines (Bose *et al.*, 1992*b*). The most efficient compound (**1**) was approximately 50- and 300-fold more efficient than the major-groove cross-linking agents cisplatin and melphalan respectively. Molecular modelling with permutations of $d(CGYGXXCYGG)_2$ suggests that spatial separation of the PBD units in compound **1** is optimal for spanning 6 bp, with a preference for 5′-PuGATCPy or 5′-PyGATCPu sequences, and that it actively recognises the embedded $d(GATC)_2$ motif (Bose *et al.*, 1992*a*). This represents an enhanced sequence recognition compared with the monomer unit DC-81 (Figure 1), which spans only three bases, only two of which are actively recognised (Thurston, 1993). H-NMR examination of a 1:1 adduct of **1** with $d(CICGATCICG)_2$ has shown that the duplex is covalently cross-linked symetrically via the minor-groove N2 positions of the guanines with minimal distortion of the DNA helix. Molecular modelling has predicted that with compound **3** the extended linker should be optimal to allow the inclusion of an extra base pair within the cross-linked sequence, and this has recently been verified in synthetic oligonucleotides (Smellie *et al.*, 1993).

This paper investigates aspects of the cellular pharmacology of this novel series of agents. Cytotoxicity was assessed in human tumour cell lines, and cross-resistance patterns studied in cell lines with characterised mechanisms

Compound 1 $n = 3$
Compound 2 $n = 4$
Compound 3 $n = 5$
Compound 4 $n = 6$

**Figure 1** Chemical structures of anthramycin, DC81 and the four C8-linked PBDs used in this study. Compound **1** is DSB-120.

Correspondence: J.A. Hartley.
Received 17 December 1993; and in revised form 3 March 1994.

of acquired resistance to the clinically used anti-tumour agents cisplatin and doxorubicin. In addition, DNA damage and its repair was assessed in cells at pharmacologically relevant doses of the agents using the technique of alkaline elution.

### Materials and methods

### *Cell culture*

The human chronic myelogenous leukaemic cell line K562 as a suspension culture was grown at 37°C in an atmosphere of 95% air/5% carbon dioxide in RPMI-1640 containing 10% heat-inactivated fetal bovine calf serum, antibiotic free.

All other lines (ovarian carcinoma, HX/62, SKOV-3, 41M, CH1, A2780; cervical carcinoma, HX/155; cisplatin-resistant sublines, 41McisR, CH1cisR, A2780cisR, HX/155cisR; doxorubicin-resistant sublines, 41MdoxR, CH1doxR) were grown as monolayer cultures at 37°C, in an atmosphere of 90% air/10% carbon dioxide in Dulbecco's modified Eagle medium (DMEM) with 10% FCS as above, 50 $\mu g\,ml^{-1}$ gentamicin, 2.5 $\mu g\,ml^{-1}$ amphotericin B, 2 mM glutamine, 10 $\mu g\,ml^{-1}$ insulin and 0.5 $\mu g\,ml^{-1}$ hydrocortisone. Establishment details and biological properties of these cell lines have been described previously (Hills *et al.*, 1989; Kelland *et al.*, 1993).

### *Drugs and drug treatment*

Compounds **1**–**4** were synthesised as previously described (Bose *et al.*, 1992*b*). Drug stock solutions were prepared in high-performance liquid chromatography (HPLC)-grade methanol immediately prior to use. The final methanol concentration used to treat cells was less than 0.5%, which did not affect cell viability. For cell cycle analysis and alkaline elution experiments, exponentially growing cells were treated with drug for 1 h at 37°C. Drug-containing medium was then removed by centrifugation and the cells resuspended in drug-free medium.

### *Cytotoxicity studies*

Drug-induced cytotoxicity was determined using the sulphorhodamine B assay as previously described (Kelland *et al.*, 1993). Cells were plated at between $5 \times 10^3$ and $1 \times 10^4$ in 96-well microtitre plates and left overnight for cells to adhere before drug treatment. After 4 days of continuous drug exposure, growth inhibition was assessed using sulphorhodamine B protein staining.

### *Cell cycle determination*

Following drug treatment and the appropriate post-incubation time in drug-free medium, aliquots of $5 \times 10^5$ K562 cells were collected by centrifugation, washed once, resuspended in ice-cold phosphate-buffered saline (PBS) (500 μl), fixed by dropwise addition of 95% ethanol (2 ml) and stored at 4°C until use. Cells were subsequently washed twice in isotonic saline and resuspended in propidium iodide solution (0.1% sodium citrate, 50 $\mu g\,ml^{-1}$ propidium iodide, 7.5 $\mu g\,ml^{-1}$ RNAse, 0.002% NP40). Cells were incubated with the stain for 45–60 min in the dark at room temperature. Cell cycle determination was carried out on a Becton Dickinson FACScan.

### *Alkaline elution analysis*

Analysis of DNA interstrand cross-link formation in K562, A2780 and A2780cisR cells was carried out using alkaline elution as described by Kohn *et al.* (1981). Logarithmically growing cells were preincubated with 0.0165 $\mu Ci\,ml^{-1}$ [$^{14}C$]thymidine (Amersham 250 μCi 5 $ml^{-1}$) overnight and then resuspended in label-free medium for 1 h. Incubation with drug was for 1 h followed by the appropriate post-incubation time in drug-free medium. Each sample ($0.6 \times 10^6$ cells in 2.5 ml) was irradiated with X-rays (450 rads) to introduce a constant frequency of single-strand breaks. Cells were then layered on 25-mm-diameter, 2 μm pore size filters (Costar Filtration Products, Nucleopore) and lysed with 5 ml of Sarcosyl lysis solution (0.2% *N*-lauroysarcosine, 2 M sodium chloride 0.04 M EDTA, pH 10). Elution was performed at pH 12.1 in the presence of 0.5 $\mu g\,ml^{-1}$ proteinase K at a constant rate of 2 $ml\,h^{-1}$. A cross-link index was calculated using the formula:

$$XLI = \sqrt{(1-R_0)/(1-R_1)} - 1$$

where $R_0$ and $R_1$ are the relative retention values at 12 h of alkaline elution for control and drug-treated samples respectively.

### *Glutathione (GSH) determination and cellular depletion by* D,L-*buthionine*-S,R-*sulphoximine (BSO)*

A2780 and A2780cisR cells ($1-2 \times 10^6$) were harvested, washed twice with cold PBS and lysed by addition of 1.5 ml of 0.6% sulphosalicyclic acid followed by a 10 min incubation at 4°C (Russo *et al.*, 1986). The supernatant was obtained for assay following centrifugation (1,200 r.p.m., 5 min, 4°C) and total GSH was determined by the enzymatic recycling method based on glutathione reductase described by Griffith (1980). GSH content was expressed as nmol $l^{-1}$ cells. Cellular depletion of glutathione was achieved with BSO (Mistry *et al.*, 1991). After plating and a 12 h attachment period, medium was aspirated and replaced with medium containing 50 μM BSO for 24 h prior to treatment with the test compound as described above.

### *Modulation of multidrug resistance by verapamil*

After a 12 h attachment period, medium containing verapamil was added to cells (CH1, CHDoxR, 41M and 41M DoxR), at a final concentration of 6 μM for 2 h prior to drug treatment as described above. The verapamil remained present in growth medium during the drug incubation.

### Results

### *Cytotoxicity of 1–4 in human ovarian and cervical carcinoma cell lines*

Cytotoxicity of compounds **1**–**4** in five human ovarian carcinoma cell lines (HX62, SKOV-3, 41M, CH1 and A2780) and one human cervical carcinoma cell line (HX155) is shown in Figure 2. These cell lines were chosen on the basis of their known pattern of inherent sensitivity to the cross-

**Figure 2** Cytotoxicity of compounds **1**–**4** in five human ovarian carcinoma cell lines (HX62, SKOV-3, 41M, CH1 and A2780) and one human cervical carcinoma cell line (HX155) as determined by the sulphorhodamine B assay. Results are the mean ± s.d. of at least three independent experiments.

linking drug cisplatin and the availability of sublines with both cisplatin and doxorubicin acquired resistance. The four compounds showed the same pattern of relative activity in the six cell lines with $IC_{50}$ values in the order $1 \leqslant 3 < 4 < 2$. This order is consistent with that previously reported in the human leukaemic cell line K562 (Bose et al., 1992b). The most potent compound (1) was 120-fold more active in the most sensitive cell line A2780 than in the least sensitive line HX155. In addition, the differential toxicity between the most active (1) and least active compound (2) was greatest in the most sensitive cell lines, CH1 and A2780 (237- and 168-fold respectively), and smallest in the least sensitive (HX155, 23-fold). For comparison, compound 1 was between 4-fold (HX155) and 80-fold (A2780) more active than cisplatin under identical conditions.

*Effect of 1 on the cell cycle*

The effect of the most potent compound (1) on the cell cycle was analysed in the human leukaemic cell line K562 following a 1 h exposure (Figure 3). The $IC_{50}$ under these conditions is 0.25 μM. A clear dose-dependent accumulation of cells in the $G_2/M$ phase of the cell cycle was evident at subtoxic concentrations of drug, and the persistence of the block was dependent on the drug dose and the time of post-incubation. Such a block is consistent with the known action of DNA-damaging and cross-linking agents (Konopa, 1988).

*DNA interstrand cross-linking in cells*

Compounds 1–4 have previously been shown to be highly efficient DNA interstrand cross-linking agents in isolated DNA (Bose et al., 1992b). The ability of compound 1 to produce interstrand cross-links in K562 cells was investigated using the technique of alkaline elution. Following a 1 h exposure to drug, a dose-dependent increase in cross-linking is observed with no evidence of DNA single-strand breakage (Figure 4a). Cross-linking increases with time up to 24 h of post-incubation, and this level of cross-linking is maintained at 48 h (Figure 4b). This is in contrast to other cross-linking agents, such as chlorambucil or cisplatin, which produce a peak of cross-linking at 6–12 h in the cell line with extensive repair evident by 24 h (data not shown). At an equimolar dose of the least active compound (2) no cross-linking was observed at any time point. Compounds 3 and 4 produced cross-links, but at a lower level than 1. Again there was no evidence of repair at 48 h (data not shown).

Figure 4 DNA interstrand cross-linking by 1 in K562 cells. a, Alkaline elution profiles of DNA from cells treated for 1 h with O (+), 0.1 μM (○, ●), 0.5 μM (□, ■) and 1 μM (△, ▲) drug followed by 4 h post-incubation in drug-free medium. Cells were either irradiated (open symbols) or unirradiated (closed symbols). b, Time course of cross-linking in K562 cells following a 1 h exposure to 0.5 μM compound 1. Results are the mean ± s.d. of at least three independent experiments.

Figure 3 Cell cycle perturbations induced by compound 1 in K562 cells. Incubations were for 1 h at the doses indicated followed by post-incubation in drug-free medium for 1–4 days.

*Effect of compounds 1–4 on cisplatin- and doxorubicin-resistant cell lines*

The cytotoxicity of all four compounds was evaluated simultaneously in a panel of both cisplatin- and doxorubicin-sensitive and acquired resistant human tumour cell lines. The resulting resistance factors ($IC_{50}$ resistant/parent) are listed in Table I. In the cisplatin-resistant lines compounds **1–4** gave similar results in that they retained activity in two of the resistant lines (41McisR and HX155cisR), retained partial activity against the CH1cisR lines, but showed total cross-resistance with cisplatin in the A2780-resistant line.

In the 41M-doxR line all four compounds showed a similar level of resistance to doxorubicin itself. With the CH1-resistant line all compounds showed some cross-resistance, but this varied from 12-fold (**2**) to 84-fold (**3**) compared with 150-fold for doxorubicin itself.

*Effect of BSO pretreatment on the cytotoxicity and DNA interstrand cross-linking by compound 1 in the cisplatin-resistant cell line A2780cisR*

Compounds **1–4** showed a similar level of resistance to cisplatin in the A2780cisR resistant cell line. As this cell line is known to have elevated glutathione, increased DNA repair of cisplatin adducts and decreased uptake of cisplatin (Behrens *et al.*, 1987), the mechanisms involved in the resistance to **1** were investigated further. Following a 1 h exposure to drug the resistant cell line was approximately 11-fold less sensitive to **1** than the parent (Table II). At 24 h post incubation the cross-link index was 2.5-fold less in the resistant line (Table II) following an equimolar dose of drug. Both a lower extent and slower rate of formation of cross-links were observed in the resistant line, with no evidence of DNA repair in either line at 38 h (Figure 5). The level of glutathione in the resistant line was almost twice that in the sensitive line (Table II). Following a 24 h non-cytotoxic pretreatment of the resistant cells with 50 μM BSO, glutathione levels were reduced to below that in the sensitive line (Table II). The level of resistance was reduced from 11-fold to 2-fold compared with the sensitive cells, and the level of cross-linking was increased 2-fold. In contrast, the level of resistance to cisplatin was only reduced 1.5-fold.

Similar results were also obtained using the intrinsically cisplatin-resistant SKOV-3 cell line, which has been shown to have levels of GSH 4-fold higher than cisplatin-sensitive ovarian carcinoma cells (Mistry *et al.*, 1991). Whereas the $IC_{50}$ value of **1** alone was 6.2 μM (2 h exposure), preincubations for 24 h with 50 μM BSO reduced the $IC_{50}$ value by 7.8-fold to 0.79 μM.

*Effect of verapamil on the cytotoxicity of 1 in doxorubicin-resistant lines*

Treatment of cells with verapamil decreased the level of resistance to **1** in both doxorubicin-resistant lines (Table III). The resistance factors were decreased by 7.6-fold and 1.7-fold in the CH1 pair and 41M pair respectively. These were somewhat lower than for doxorubicin itself, which decreased by 17.2-fold and 3.1-fold respectively.

### Discussion

The ranking order of potency of compounds **1–4** was the same in the five human ovarian and one cervical tumour cell lines studied *in vitro*, with the differential being greatest in the most sensitive cell lines. This same order was previously reported in three murine lines and the human leukaemic

**Figure 5** DNA interstrand cross-link formation in A2780 (open symbols) or A2780cisR cells (closed symbols) following a 1 h exposure to 0.5 μM compound **1**. Results are mean ± s.d. of at least three experiments.

**Table I** Resistance factors for compounds **1–4** in cisplatin- and doxorubicin-resistant ovarian and cervical cancer cell lines

| *Cell line pair* | | *Resistance factor[a] ($IC_{50}$ resistant/parent)* *1* | *2* | *3* | *4* |
|---|---|---|---|---|---|
| | Cisplatin | | | | |
| 41M/41McisR | ~5 | 0.6 | 0.7 | 0.6 | 0.8 |
| CH1/CH1cisR | ~7 | 2.3 | 2.1 | 2.3 | 2.6 |
| A2780/A2780cisR | ~11 | 13.8 | 8.6 | 10.2 | 10.8 |
| HX155/HX155cisR | ~9 | 0.4 | 0.9 | 0.4 | 0.5 |
| | Doxorubicin | | | | |
| 41M/41MdoxR | ~7.7 | 4.2 | 3.3 | 5.2 | 6.3 |
| CH1/CH1doxR | ~150 | 65 | 12 | 84 | 31 |

[a]Mean of three independent experiments. Drug exposure was continuous.

**Table II** Cytotoxicity, glutathione content and DNA interstrand cross-linking in A2780 cells and the cisplatin-resistant subline with and without depletion of GSH by BSO

| *Cell line* | *$IC_{50}$[a]* 1 | *GSH[b]* *(nmol $10^{-6}$ cells)* | *Cross-link index[c]* | *$IC_{50}$ cisplatin* |
|---|---|---|---|---|
| A2780 | 0.1 ± 0.02 | 5.8 ± 1 | 0.5 ± 0.1 | 0.9 ± 0.2 |
| A2780cisR | 1.1 ± 0.2 | 10.3 ± 1.5 | 0.2 ± 0.1 | 7.8 ± 0.9 |
| A2780cisR + BSO | 0.2 ± 0.07 | 2.8 ± 1.1 | 0.4 ± 0.01 | 5.3 ± 1 |

[a]One hour exposure to drug, mean ± s.d. of at least three independent determinations. [b]Mean ± s.d. of three determinations. [c]Cross-link index at 24 h following a 1 h exposure to 0.5 μM drug. Mean ± s.d. of three independent experiments.

**Table III** Effect of verapamil on the cytotoxicity of 1 in the doxorubicin (DOX)-resistant lines

| | *Resistance factor*[a] | | | |
|---|---|---|---|---|
| | *Dox* | | 1 | |
| *Cell line pair* | *– ver* | *+ ver*[b] | *– ver* | *+ ver* |
| 41M/41MdoxR | 7.7 ± 1.3 | 2.5 ± 0.66 | 3.7 ± 0.8 | 2.2 ± 0.2 |
| CH1/CH1doxR | 150 | 8.7 | 80 | 10.5 |

[a]Mean of three independent experiments. [b]Cells were treated with 6 μM verapamil (ver) for 2 h prior to drug treatment, and during drug exposure. This dose of verapamil resulted in no loss of viability in control cells.

K562 line (Bose *et al.*, 1992*b*). The most potent compound (1) was found to be between 4- and 80-fold more active than cisplatin in these cell lines.

The study of Bose *et al.* (1992*a*) demonstrated that the ranking order of cytotoxicity in tumour cell lines was the same as DNA interstrand cross-linking ability as measured in plasmid DNA using an agarose gel-based technique. These data strongly suggested that the cytotoxicity of this class of compounds is associated with the formation of bifunctional lesions on DNA. The dose- and time-dependent blockage of cells in the $G_2/M$ phase of the cell cycle observed in the present study is characteristic of DNA-damaging agents (Konopa, 1988), and, in particular, DNA interstrand cross-linking agents such as melphalan (Brox *et al.*, 1980) and cisplatin (Sorenson & Eastman, 1988). The present study also demonstrated through alkaline elution that compound 1 is a highly efficient DNA interstrand cross-linking agent in cells. In addition, with compounds 1–4 the level of cross-linking in cells at an equimolar dose reflected the order of cytotoxicities.

DNA interstrand cross-linking has been shown to correlate with cytotoxicity for different classes of bifunctional alkylating agents, including nitrogen mustards (Zwelling *et al.*, 1981; O'Connor & Kohn, 1990; Sunters *et al.*, 1992), chloroethylnitrosoureas (Erickson *et al.*, 1980*a*,*b*), dimethane sulphonates of the busulphan series (Bedford & Fox, 1989) and cisplatin (Zwelling *et al.*, 1979*a*, 1981). It is generally assumed that an interstrand cross-link, if not repaired, would interfere with the process of DNA replication. DNA repair has been shown to be an important determinant of sensitivity to many cross-linking agents, and the mechanisms involved are being elucidated (Burt *et al.*, 1991). However, the cross-linking in cells by 1 showed some important differences compared with more classical agents such as melphalan and cisplatin, whose kinetics of cross-link formation and removal have been found to be generally similar. After treatment of cells with melphalan at doses equivalent to those used in the present study, maximim cross-linking is observed at 4–6 h with extensive evidence of repair at 24 h post treatment (Zwelling *et al.*, 1979*b*; O'Connor & Kohn, 1990). Similarly, treatment of cells with a wide range of doses of cisplatin results in a peak of cross-linking at 6–12 h (Zwelling *et al.*, 1978). The removal of cross-links is faster after treatment with higher doses of cisplatin (Pera *et al.*, 1981). In contrast, the cross-linking observed in the present study by 1 continued to increase to a peak at 24 h following a 1 h drug treatment with no evidence of removal at 36 or 48 h.

The apparent lack of repair of 1-induced cross-links may be due to an equilibrium reached between cross-link formation and repair in the cells. Alternatively, it may be that these lesions in the minor groove of DNA are not recognised by the same repair enzyme complexes that recognise and repair melphalan and cisplatin cross-links in the major groove of DNA. Previous nuclear magnetic resonance (NMR) and molecular modelling studies have demonstrated that the complex of 1, with its optimum binding sequence of 5'-PuGATCPy and the drug cross-linked symmetrically via the N2-guanine positions, provides minimal distortion of the DNA helix (Bose *et al.*, 1992*b*). Such a complex may then not be recognised by a DNA repair mechanism which is scanning for distortion of the DNA.

Studies in the characterised pairs of sensitive and resistant cell lines were undertaken to assess the factors which may affect the sensitivity of cells to 1. The results obtained would appear to be related to the major mechanism associated with the resistance of each line. 41McisR and HX155cisR are resistant mainly because of decreased uptake of cisplatin, while CH1cisR is resistant because of enhanced DNA repair of platinum adducts (Kelland *et al.*, 1992; Mellish *et al.*, 1993). No cross-resistance to 1 was observed in the former lines, indicating that uptake of cisplatin and 1 are by different mechanisms. Compound 1 retained partial activity against CH1cisR, suggesting that some elements of the enhanced repair of platinum adducts may be involved in repair of 1. The A2780cisR line has been shown to possess a combination of elevated glutathione, increased DNA repair and decreased uptake (Behrens *et al.*, 1987). With this line, 1 showed a similar (14-fold) level of resistance to cisplatin. By comparison with the 41McisR and HX155cisR lines, drug uptake is not thought to be involved in the resistance to 1. Similarly, enhanced DNA repair is not thought to play a major role since no evidence of repair of 1-induced cross-links was observed in this cell line at 48 h. The partial reversal of the resistance to 1 in the A2780cisR line by GSH depletion using BSO (with resultant increase in interstrand cross-linking) indicates that resistance is largely due to inactivation of the drug by GSH binding in cells. Further evidence for this is provided by the comparative $IC_{50}$ data for the HX62 (0.3 μM) *vs* 41 M (0.04 μM) cell lines, as previous studies have shown a 4-fold higher GSH level in the HX62 cells (Mistry *et al.*, 1991). Previous studies have already shown that PBDs react with thiol-containing nucleophiles such as thiophenol to give stable covalent adducts (Morris *et al.*, 1990; Morris, 1992).

Both cell lines with acquired resistance to doxorubicin (41MdoxR) showed full or partial cross-resistance to 1. High levels of cross-resistance have also been observed to vinblastine, colchicine and taxol in these cell lines, which possess elevated levels of p170 glycoprotein. Compound 1 is, therefore, a substrate for this multidrug efflux mechanism, which was confirmed by the partial reversal of resistance in the presence of the calcium channel blocker verapamil.

Compound 1 is, therefore, the most cytotoxic compound in a series of rationally designed PBD-based sequence-selective DNA minor-groove cross-linking agents. It is a highly efficient interstrand cross-linking agent in cells, and the cross-links appear to be difficult to repair. Levels of glutathione and p170-glycoprotein clearly determine the sensitivity of cells to 1. Further investigation will determine how much these factors affect the efficacy of 1 *in vivo*.

M.S. thanks SERC for a studentship. This work was funded in part by the Cancer Research Campaign.

### References

BEDFORD, P. & FOX, B.W. (1983). DNA-DNA interstrand crosslinking by dimethanesulphonic acid esters. *Biochem. Pharmacol.*, **32**, 2297-2301.

BEHRENS, B.C., HAMILTON, T.C., MASUDA, H., GROTZINGER, K.R., WHANG-PENG, J., LOUIE, K.G., KNUTSEN, T., MCKOY, W.M., YOUNG, R.C. & OZOLS, R.F. (1987). Characterization of a *cis*-diamminedichloroplatinum (II)-resistant human ovarian cancer cell line and its use in evaluation of platinum analogues. *Cancer Res.*, **47**, 414-418.

BOSE, D.S., THOMPSON, A.S., CHING, J., HARTLEY, J.A., BERARDINI, M.D., JENKINS, T.C., NEIDLE, S., HURLEY, L.H. & THURSTON, D.E. (1992*a*). Rational design of a highly efficient irreversible DNA interstrand cross-linking agent based on the pyrrolobenzodiazepine ring system. *J. Am. Chem. Soc.*, **114**, 4939-4941.

BOSE, D.S., THOMPSON, A.S., SMELLIE, M., BERARDINI, M.D., HARTLEY, J.A., JENKINS, T.C., NEIDLE, S. & THURSTON, D.E. (1992*b*). Effect of linker length on DNA binding affinity, cross-linking efficiency and cytotoxicity of C8-linked pyrrolobenzodiazepine dimers. *J. Chem. Soc. Chem. Commun.*, **14**, 1518-1520.

BROX, L.W., GOWANS, B. & BELCH, A. (1980). L-phenylalanine mustard (melphalan) uptake and cross-linking in the RPMI 6410 human lymphoblastoid cell line. *Cancer Res.*, **40**, 1169-1172.

BURT, R.K., POIRIER, M.C., LINK, C.J. & BOHR, V.A. (1991). Antineoplastic drug resistance and DNA repair. *Ann. Oncol.*, **2**, 325-334.

ERICKSON, L.C., BRADLEY, M.O., DUCORE, J.M., EWIG, R.A.G. & KOHN, K.W. (1980*a*). DNA crosslinking and cytotoxicity in normal and SV40 transformed human cells treated with antitumour nitrosoureas. *Proc. Natl Acad. Sci. USA*, **77**, 467-471.

ERICKSON, L.C., LAURENT, G., SHARKEY, N.A. & KOHN, K.W. (1980*b*). DNA crosslinking and monoadduct repair in nitrosourea-treated human tumour cells. *Nature*, **288**, 727-729.

GRIFFITH, O.W. (1980). Determination of glutathione and glutathione disulfide using glutathione reductase and 2-vinylpyridine. *Anal. Biochem.*, **106**, 207-212.

HERTZBERG, R.P., HECHT, S.M., REYNOLDS, V.L., MOLINEUX, I.J. & HURLEY, L.H. (1986). DNA sequence specificity of the pyrrolo[1,4]benzodiazepine antitumor antibiotics. Methidiumpropyl-EDTA-iron(II) footprinting analysis of DNA binding sites for anthramycin and related drugs. *Biochemistry*, **25**, 1249-1258.

HILLS, C.A., KELLAND, L.R., ABEL, G., SIRACKY, J., WILSON, A.P. & HARRAP, K.R. (1989). Biological properties of ten human ovarian carcinoma cell lines: calibration *in vitro* against four platinum complexes. *Br. J. Cancer*, **59**, 527-534.

HURLEY, L.H. & THURSTON, D.E. (1984). Pyrrolo[1,4]benzodiazepine antitumor antibiotics; chemistry, interactions with DNA, and biological implications. *Pharm. Res.*, **2**, 52-59.

HURLEY, L.H., RECK, T., THURSTON, D.E., LANGLEY, D.R., HOLDEN, K.G., HERTZBERG, R.P., HOOVER, J.R., GALLAGHER Jr, G., FAUCETTE, L.F., MONG, S.M. & JOHNSON, R.K. (1988). Pyrrolo(1,4)benzodiazepine antitumor antibiotics: relationship of DNA alkylation and sequence specificity to the biological activity of natural and synthetic compounds. *Chem. Res. Toxicol.*, **1**, 258-268.

KELLAND, L.R., MISTRY, P., ABEL, G., LOH, S.Y., O'NEILL, C.F., MURRER, B.A. & HARRAP, K.R. (1992). Mechanism-related circumvention of acquired *cis*-diamminedichloroplatinum (II) resistance using two pairs of human ovarian carcinoma cell lines by ammine/amine platinum (IV) dicarboxylates. *Cancer Res.*, **52**, 3857-3864.

KELLAND, L.R., ABEL, G., MCKEAGE, M.J., JONES, M., GODDARD, P.M., VALENTI, M., MURRER, B.A. & HARRAP, K.R. (1993). Preclinical antitumor evaluation of bis-acetato-ammine-dichloro-cyclhexylamine platinum (IV): an orally active platinum compound. *Cancer Res.*, **53**, 2581-2586.

KOHN, K.W., EWIG, R.A.G., ERICKSON, L.C. & ZWELLING, L.A. (1981). Measurement of strand breaks and cross-links by alkaline elution. In *DNA Repair: A Laboratory Manual of Research Procedures*, Friedberg, E.C. & Hanawalt, P.C. (eds) pp. 379-402. Marcel Dekker: New York.

KONOPA, J. (1988). G2 block induced by DNA crosslinking agents and its possible consequences. *Biochem. Pharmacol.*, **37**, 2303-2309.

MELLISH, K.J., KELLAND, L.R. & HARRAP, K.R. (1993). *In vitro* platinum drug chemosensitivity of human cervical squamous cell carcinoma cell lines with intrinsic and acquired resistance to cisplatin. *Br. J. Cancer*, **68**, 240-250.

MISTRY, P., KELLAND, L.R., ABEL, G., SIDHAR, S. & HARRAP, K.R. (1991). The relationships between glutathione, glutathione-S-transferase and cytotoxicity of platinum drugs and melphalan in eight human ovarian carcinoma cells lines. *Br. J. Cancer*, **64**, 215-220.

MORRIS, S.J. (1992). Design, synthesis and evaluation of a sequence-selective DNA-cleaving agent based on the pyrrolobenzodiazepine ring system. University of Portsmouth. PhD thesis.

MORRIS, S.J., THURSTON, D.E. & NEVELL, T.G. (1990). Evaluation of the electrophilicity of DNA-binding pyrrolo[2,1-c][1,4] benzodiazepines by HPLC. *J. Antibiotics*, **43**, 1286-1292.

O'CONNOR, P.M. & KOHN, K.W. (1990). Comparative pharmacokinetics of DNA lesion formation and removal following treatment of L1210 cells with nitrogen mustards. *Cancer Commun.*, **2**, 387-394.

PERA, M.F., RAWLINGS, C.J., SHACKLETON, J. & ROBERTS, J.J. (1981). Quantitative aspects of the formation and loss of DNA interstrand cross-links in Chinese hamster cells following treatment with *cis*-diamminedichloro-platinum (11) (cisplatin). 2. Comparison of results from alkaline elution, DNA renaturation and DNA sedimentation. *Biochim. Biophys. Acta*, **655**, 152-166.

REMERS, W.A. (1988). Pyrrolobenzodiazepines. In *The Chemistry of Antitumour Antibiotics*, pp. 28-92. John Wiley: Chichester.

RUSSO, A., DE GRAFF, W., FRIEDMAN, N. & MITCHELL, J.B. (1986). Selective modulation of glutathione levels in human normal versus tumour cells and subsequent differential response to chemotherapy drugs. *Cancer Res.*, **46**, 2845-2848.

SMELLIE, M., BERARDINI, M.D., BOSE, D.S., THOMPSON, A.S., THURSTON, D.E. & HARTLEY, J.A. (1993). Molecular pharmacology of novel linked anthramycin-based sequence selective crosslinking agents. *Br. J. Cancer*, **67**, (Suppl. XX), 8.

SORENSON, C.M. & EASTMAN, A. (1988). Mechanism of *cis*-diamminedichloroplatinum (II)-induced cytotoxicity: role of G2 arrest and DNA double strand breaks. *Cancer Res.*, **48**, 4484-4488.

SUNTERS, A., SPRINGER, C.J., BAGSHAWE, K.D., SOUHAMI, R.L. & HARTLEY, J.A. (1992). The cytotoxicity, DNA crosslinking ability and DNA sequence selectivity of the alanine mustards melphalan, chlorambucil and 4-[bis](2-chloroethyl)amino] benzoic acid. *Biochem. Pharmacol.*, **41**, 59-64.

THURSTON, D.E. (1993). Advances in the study of pyrrolo[2,1-c][1,4]-benzodiazepine (PBD) antitumour antibiotics. In *Molecular Aspects of Anticancer Drug-DNA Interactions*, Neidle, S. & Waring, M.J. (eds) pp. 54-88. Macmillan Press: London.

ZWELLING, L.A., KOHN, K.W., ROSS, W.E., EWIG, R.A.G. & ANDERSON, T. (1978). Kinetics of formation and disappearance of a DNA cross-linking effect in mouse leukemia L1210 cells treated with *cis*- and *trans*-platinum (II) diamminedichloroplatinum. *Cancer Res.*, **38**, 1762-1768.

ZWELLING, L.A., ANDERSON, T. & KOHN, K.W. (1979*a*). DNA-protein and DNA interstrand cross-linking by *cis*- and *trans*-platinum (II) diamminedichloride in L1210 mouse leukemia cells and relation to cytotoxicity. *Cancer Res.*, **39**, 365-369.

ZWELLING, L.A., FILIPSKI, J. & KOHN, K.W. (1979*b*). Effect of thiourea on survival and DNA cross-link formation in cells treated with platinum (II) complexes, L-phenylalanine mustard, and bis(2-chloroethyl)methylamine. *Cancer Res.*, **39**, 4989-4995.

ZWELLING, L.A., MICHAELS, S., SCHWARTZ, H., DOBSON, P.P. & KOHN, K.W. (1981). DNA cross-linking as an indicator of sensitivity and resistance of mouse L1210 leukemia to *cis*-diamminedichloroplatinum (II) and L-phenylalanine mustard. *Cancer Res.*, **41**, 640-649.